# Diabetic silkworms for evaluation of therapeutically effective drugs against type II diabetes

**DOI:** 10.1038/srep10722

**Published:** 2015-05-29

**Authors:** Yasuhiko Matsumoto, Masaki Ishii, Yohei Hayashi, Shinya Miyazaki, Takuya Sugita, Eriko Sumiya, Kazuhisa Sekimizu

**Affiliations:** 1 Laboratory of Microbiology, Graduate School of Pharmaceutical Sciences, The University of Tokyo, 7-3-1 Hongo, Bunkyo-ku, Tokyo 111-0033, Japan

## Abstract

We previously reported that sugar levels in the silkworm hemolymph, i.e., blood, increase immediately (within 1 h) after intake of a high-glucose diet, and that the administration of human insulin decreases elevated hemolymph sugar levels in silkworms. In this hyperglycemic silkworm model, however, administration of pioglitazone or metformin, drugs used clinically for the treatment of type II diabetes, have no effect. Therefore, here we established a silkworm model of type II diabetes for the evaluation of anti-diabetic drugs such as pioglitazone and metformin. Silkworms fed a high-glucose diet over a long time-period (18 h) exhibited a hyperlipidemic phenotype. In these hyperlipidemic silkworms, phosphorylation of JNK, a stress-responsive protein kinase, was enhanced in the fat body, an organ that functionally resembles the mammalian liver and adipose tissue. Fat bodies isolated from hyperlipidemic silkworms exhibited decreased sensitivity to human insulin. The hyperlipidemic silkworms have impaired glucose tolerance, characterized by high fasting hemolymph sugar levels and higher hemolymph sugar levels in a glucose tolerance test. Administration of pioglitazone or metformin improved the glucose tolerance of the hyperlipidemic silkworms. These findings suggest that the hyperlipidemic silkworms are useful for evaluating the hypoglycemic activities of candidate drugs against type II diabetes.

Diabetes, a disease characterized by chronic hyperglycemia and impaired glucose tolerance, has many adverse effects, such as retinopathy, renal disease, and peripheral neuropathy^1^. Insulin, a peptide hormone, has potent hypoglycemic activity, and is used clinically to treat diabetes^2^. The number of patients with type II diabetes, which is associated with insulin resistance, is increasing worldwide^3^,^4^. Drugs that ameliorate insulin resistance are often prescribed for patients with type II diabetes, but drug-resistance and side effects are major concerns^5^,^6^. Thus, the development of novel anti-diabetic drugs is urgently needed.

Plasma glucose levels are regulated by tissue uptake and metabolism throughout the body. Thus, evaluation of anti-diabetic drugs requires the use of an appropriate animal model in which plasma glucose levels can be quantitatively determined^7^. Mammals such as mice and rats are used to screen anti-diabetic drugs, but the high cost of maintaining a large number of animals as well as the ethical issues surrounding animal welfare are serious drawbacks of these models. Experiments using animal models should be performed following the 3Rs concept (Replacement, Reduction, and Refinement), which is an internationally recognized principle for responsibly conducting animal experiments^8^ for drug development. The establishment of new invertebrate animal models of diabetes is consistent with the idea of “Replacement”. To overcome the high cost and ethical aspects of using mammalian animal models for drug development, several disease models have been established using invertebrate model animals such as fruit flies (*Drosophila melanogaster*) and nematodes (*Caenorhabditis elegans*)^9^,^10^,^11^,^12^,^13^,^14^,^15^,^16^. The small body sizes of these animals, however, make it difficult to inject test samples and collect hemolymph.

We have proposed the use of silkworms (*Bombyx mori*) as an animal model for the primary screening of anti-diabetic drugs, as silkworms exhibit slow moving behavior and their larger body size is adequate for injecting test samples. Hemolymph sampling methods for determining sugar levels in the silkworm are well established^17^. Furthermore, silkworm infection models are useful for quantitative evaluation of the therapeutic activities of anti-bacterial, anti-fungal, and anti-viral drugs^18^,^19^,^20^,^21^. We also observed common pharmacokinetic features of chemicals in silkworms and mammals^22^. These properties of silkworms are highly advantageous for the biochemical experiments that are necessary for evaluating the therapeutic effects of candidate drugs.

In both invertebrates and mammalian animals, blood sugar levels are regulated by the insulin-signaling pathway. In silkworms, bombyxin acts as an insulin homolog^23^. We previously reported the usefulness of hyperglycemic silkworms, established by feeding a high-glucose diet for less than 1 h, for evaluation of the hypoglycemic effects of human insulin and 5-aminoimidazole-4-carboxamide-1-beta-D-ribofuranoside (AICAR), an AMP activated protein kinase (AMPK) activator^24^. We also identified a compound with hypoglycemic activity in an herbal medicine using hyperglycemic silkworms^24^. Based on these findings, we proposed that silkworms are suitable for screening large numbers of compounds to identify candidate drugs with hypoglycemic activities.

While there are a number of advantages of using silkworms for screening compounds with hypoglycemic activity, the previously reported model^24^ was problematic for evaluating drugs against type II diabetes due to the lack of evidence that the hyperglycemic silkworms exhibited impaired glucose tolerance, which is a characteristic feature of type II diabetes. Furthermore, administration of pioglitazone or metformin, drugs used to treat type II diabetes, did not decrease the hemolymph sugar levels of the hyperglycemic silkworms (Supplementary Fig. 1). Therefore, the previous method of preparing a hyperglycemic silkworm model by short-term feeding of a high-glucose diet is not a suitable model of type II diabetes in humans. In the present study, we aimed to establish a type II diabetes model using silkworms to evaluate anti-diabetic drugs. In mammals such as mice, rats, and humans, continuous intake of a high-calorie diet leads to obesity and insulin resistance^1^. Therefore, we hypothesized that silkworms fed a high-glucose diet over a long time-period might exhibit phenotypes of type II diabetes.

In this paper, we report that silkworms fed a high-glucose diet over a long time-period (18 h) exhibited abnormal lipid metabolism, insulin resistance, and impaired glucose tolerance, and that injection of pioglitazone and metformin decreased fasting hemolymph sugar levels in silkworms. This is the first report that the therapeutic effects of drugs for type II diabetes can be evaluated using an invertebrate animal.

## Results

### Hyperlipidemic phenotypes of silkworms fed a high-glucose diet for a long time-period, 18 h

**Figure 1:**
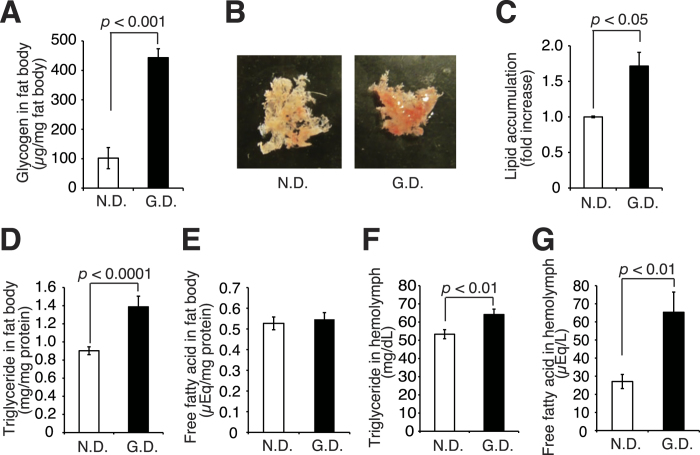
Changes in the amounts of glycogen, triglycerides, and free fatty acids in the fat body and hemolymph of silkworms fed a high-glucose diet for 18 h. (**A**–**C**) Silkworms were fed a normal diet (N.D.) or a diet containing 10% (w/w) glucose (G.D.) for 18 h, and then the silkworm fat bodies were isolated. (**A**) The amounts of glycogen in the silkworm fat bodies were determined using the anthrone-sulfuric acid method (n = 7/group). (**B**) Fat bodies were stained with Oil Red O. (**C**) Quantification of Oil Red O extracted from the stained fat bodies (n = 3/group). (**D**–**G**) Silkworms were fed a normal diet (N.D.) or a diet containing 10% (w/w) glucose (G.D.) for 18 h, and the amounts of triglycerides (**D**,**F**) and free fatty acids (**E**,**G**) in the silkworm fat bodies and hemolymph were determined (n = 8/group). Data represent mean ± SEM. Significant differences between groups were evaluated using Student’s *t*-test.

In the present study, we determined the conditions in which silkworms exhibit the phenotypes of impaired glucose tolerance and insulin resistance, characteristic features of type II diabetes. Obese mice, which exhibit insulin resistance induced by continuous intake of a high-fat diet, accumulate glycogen and triglycerides in the liver and free fatty acids in the blood^25^. Therefore, we first examined glycogen and lipid accumulation in the fat bodies and hemolymph of silkworms fed a diet containing 10% glucose (high-glucose diet) over a long time-period (18 h). The amount of glycogen in the fat bodies of silkworms fed a high-glucose diet was 4-fold higher than that of normal diet-fed silkworms (Fig. 1A). Fat bodies isolated from silkworms fed a high-glucose diet were more strongly stained with Oil red O than those from silkworms fed a normal diet (Fig. 1B,C). The amount of triglycerides in the fat bodies of silkworms fed a high-glucose diet was 1.4-fold higher than that of normal diet-fed silkworms (Fig. 1D). On the other hand, the amount of free fatty acids in the fat bodies of the high-glucose diet-fed silkworms was not significantly different from that of the normal diet-fed silkworms (Fig. 1E). The amounts of both triglycerides and free fatty acids in the hemolymph of silkworms fed a high-glucose diet were higher than those of normal silkworms (Fig. 1F,G). These findings suggest that feeding on a high-glucose diet for a long time-period leads to abnormal lipid metabolism in silkworms. We named this silkworm model the “hyperlipidemic silkworm”.

### Stimulation of c-Jun N-terminal kinase (JNK) phosphorylation in the fat bodies of the hyperlipidemic silkworms

**Figure 2:**
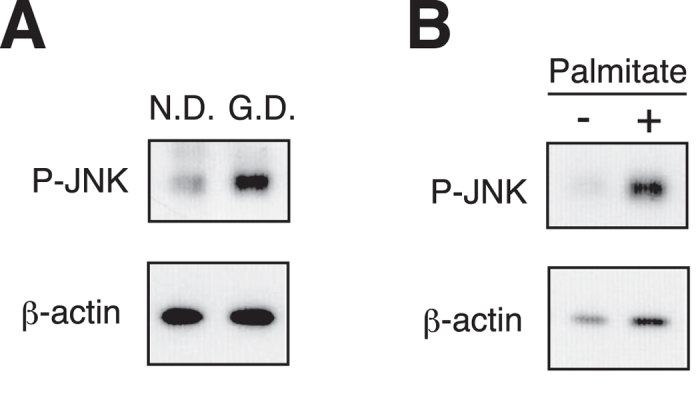
Activation of JNK phosphorylation in the fat body cells of hyperlipidemic silkworms. (**A**) Silkworms were fed a normal diet (N.D.) or a diet containing 10% (w/w) glucose (G.D.) for 18 h, and the fat bodies of the silkworms were isolated. Phosphorylated JNK and β-actin were determined by Western blot analysis. (**B**) Silkworms were fed a normal diet (N.D.) for 18 h, and then the silkworm fat bodies were isolated. Fat bodies were cultured in Grace’s insect medium (Life technologies) with 0.2% Bovine serum albumin (BSA) or palmitate (final conc. 500 μM and 0.2% BSA) at 27 °C for 1 h. Phosphorylated JNK and β-actin were determined by Western blot analysis. Samples were loaded in the same gel. Cropped blots were used. Full-length blots are presented in Supplementary Fig. 6.

Increases in the amounts of triglycerides and free fatty acids in blood and tissues, such as liver and adipose tissues, are considered to cause insulin resistance in the cells of these compartments^26^,^27^,^28^. Excess amounts of free fatty acids in the blood leads to JNK activation in tissues like the liver, and causes insulin resistance through inhibition of the insulin-signaling pathway^27^. We examined the JNK phosphorylation level in the fat bodies of the hyperlipidemic silkworms and found that phospho-JNK was increased in the hyperlipidemic silkworms compared with the normal silkworms (Fig. 2A). We further examined the effect of palmitate, a free fatty acid, against the phosphorylation of JNK in the fat bodies of normal silkworms using an *in vitro* tissue culture system. The amount of phosphorylated JNK in the fat body was increased by palmitate treatment (Fig. 2B). These findings suggest that hyperlipidemia stimulates JNK phosphorylation in the fat bodies of silkworms.

### Insulin resistance in the hyperlipidemic silkworms

**Figure 3:**
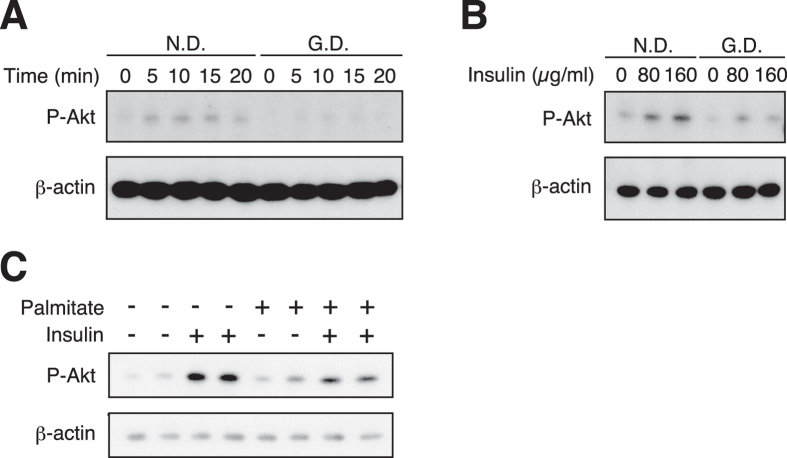
Decrease in Akt phosphorylation facilitated by human insulin in cells of fat body in the hyperlipidemic silkworm. (**A**,**B**) Silkworms were fed a normal diet (N.D.) or a diet containing 10% (w/w) glucose (G.D.) for 18 h, and then the silkworm fat bodies were isolated. (**A**) Fat bodies were cultured in Grace’s insect medium with or without human insulin (final conc. 160 μg/ml) at 27 °C for 0-20 min. (**B**) Fat bodies were cultured in Grace’s insect medium with or without human insulin (final conc. 0-160 μg/ml) at 27 °C for 15 min. (**C**) Silkworms were fed a normal diet (N.D.) for 18 h, and then the fat bodies were isolated. Fat bodies were cultured in Grace’s insect medium with 0.2% BSA or palmitate (final conc. 500 μM) with 0.2% BSA at 27 °C for 1 h. Human insulin (final conc. 160 μg/ml) or Grace’s insect medium were added to the culture medium, and the fat bodies were further incubated at 27 °C for 20 min. Phosphorylated Akt and β-actin were determined by Western blot analysis. Samples were loaded in the same gel. Cropped blots were used. Full-length blots are presented in Supplementary Fig. 6.

Cells of the liver and adipose tissues of diabetic mammals, such as mice and rats, exhibit reduced capacities to respond to insulin^27^,^28^. Therefore, we hypothesized that fat body cells of the hyperlipidemic silkworms might also exhibit insulin resistance. We previously reported that Akt phosphorylation in the cells of isolated fat bodies is stimulated by treatment with human insulin in an *in vitro* tissue culture system^24^. In the present study, we isolated the fat bodies from the hyperlipidemic silkworms and normal silkworms and compared their insulin sensitivities in the *in vitro* tissue culture system. We found that while insulin treatment enhanced Akt phosphorylation in fat bodies isolated from normal silkworms, this effect was smaller in fat bodies from the hyperlipidemic silkworms (Fig. 3A,B). We further examined the effect of palmitate against Akt phosphorylation in the fat bodies of normal silkworms with or without the co-treatment of human insulin. The amount of phosphorylated Akt in the silkworm fat bodies treated with palmitate was decreased under the insulin-treated condition (Fig. 3C). On the other hand, the amount of phosphorylated Akt in the fat bodies of silkworms was not changed by adding glucose to the culture medium to a final concentration of 350 mg/dL, which matches the glucose level in the hemolymph of hyperlipidemic silkworms (Supplementary Fig. 2). These findings suggest that the cells in the fat body of the hyperlipidemic silkworms are insulin-resistant.

### Impaired glucose tolerance of the hyperlipidemic silkworms

**Figure 4:**
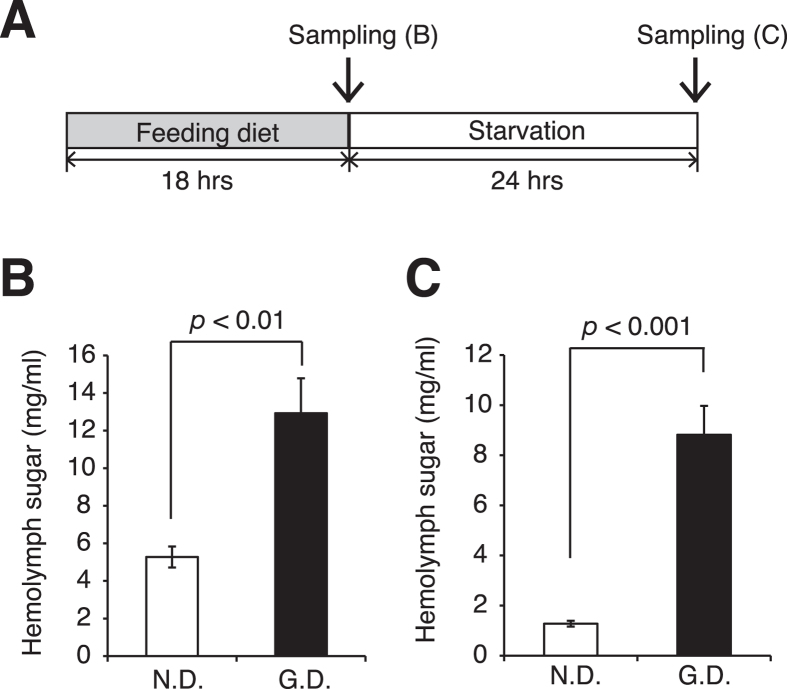
Increases in fasting hemolymph sugar levels in the hyperlipidemic silkworm. (**A**) Experimental design. (**B**) Silkworms were fed a normal diet (N.D.) or a diet containing 10% (w/w) glucose (G.D.) for 18 h. Hemolymph sugar levels of the silkworms were determined using the phenol-sulfuric acid method. (**C**) Silkworms were fed a normal diet (N.D.) or a diet containing 10% (w/w) glucose (G.D.) for 18 h, and then the diets were removed. Silkworm hemolymph sugar levels were determined after starvation for 24 h (n = 5-7/group). Data represent mean ± SEM. Significant differences between groups were evaluated using Student’s *t*-test.

**Figure 5:**
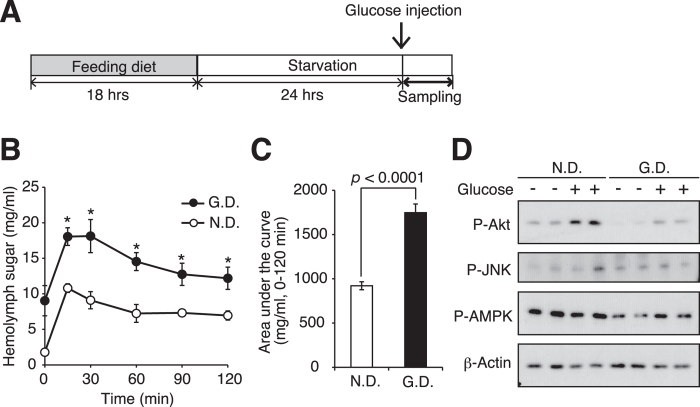
Impaired glucose tolerance of the hyperlipidemic silkworm. (**A**) Experimental design. (**B**) Silkworms were fed a normal diet (N.D.) or a diet containing 10% (w/w) glucose (G.D.) for 18 h, and then the diets were removed. After incubation for 24 h, 100 μl of glucose (75 mg/ml) was injected into the silkworm hemolymph and the hemolymph sugar levels were determined at 0, 15, 30, 60, 90, and 120 min (n = 5/group). Data represent mean ± SEM. Asterisks indicate statistical significance based on Student’s *t*-test (*p* < 0.05). (**C**) Area under the curves of hemolymph sugar levels in Fig. 5B were calculated (n = 5/group). Data represent mean ± SEM. Significant differences between groups were evaluated using Student’s *t*-test. (**D**) Silkworms were fed a normal diet (N.D.) or a diet containing 10% (w/w) glucose (G.D.) for 18 h, and then the diets were removed. After 24 h, 100 μl of glucose (75 mg/ml) was injected into the silkworm hemolymph and the silkworms were further kept for 30 min. Phosphorylated Akt, phosphorylated JNK, phosphorylated AMPK and *β* -actin in the fat bodies were determined by Western blot analysis. Samples were loaded in the same gel. Cropped blots were used. Full-length blots are presented in Supplementary Fig. 6.

Insulin resistance caused by continuous intake of a high-calorie diet leads to impaired glucose tolerance in mammals^7^,^28^. Thus, we tested whether the hyperlipidemic silkworms exhibit impaired glucose tolerance. Silkworms were fed a high-glucose diet for 18 h, and then further reared without food for 24 h (Fig. 4A). After feeding on the high-glucose diet for 18 h, the level of total sugar in the hemolymph of the silkworms fed the high-glucose diet was 2.4-fold higher than that of the normal silkworms (Fig. 4B). Furthermore, we determined the level of glucose in hemolymph of the silkworms (Supplementary Fig. 3). The higher total sugar levels in the hemolymph of the silkworms fed the high-glucose diet persisted even after 24 h of starvation (Fig. 4C). Next, we performed a glucose tolerance test on the silkworms according to the protocol shown in Fig. 5A. Hemolymph sugar levels were significantly higher at 15, 30, 60, 90, and 120 min after injecting glucose in the hyperlipidemic silkworms compared with the normal silkworms (Fig. 5B). The area under the curve of the plots of the hyperlipidemic silkworms was 2-fold greater than that of the normal silkworms (Fig. 5C). We tested whether the JNK, Akt, and AMPK signaling pathways are altered in the fat bodies of the hyperlipidemic silkworms when injected with glucose. The amount of phosphorylated Akt in the fat bodies of hyperlipidemic silkworms injected with glucose was decreased compared to normal silkworms (Fig. 5D). On the other hand, the amounts of phosphorylated JNK and AMPK in the fat bodies of hyperlipidemic silkworms injected with glucose were not altered (Fig. 5D). These findings suggest that the hyperlipidemic silkworms exhibit impaired glucose tolerance.

### Improved glucose tolerance in hyperlipidemic silkworms administered pioglitazone or metformin

**Figure 6:**
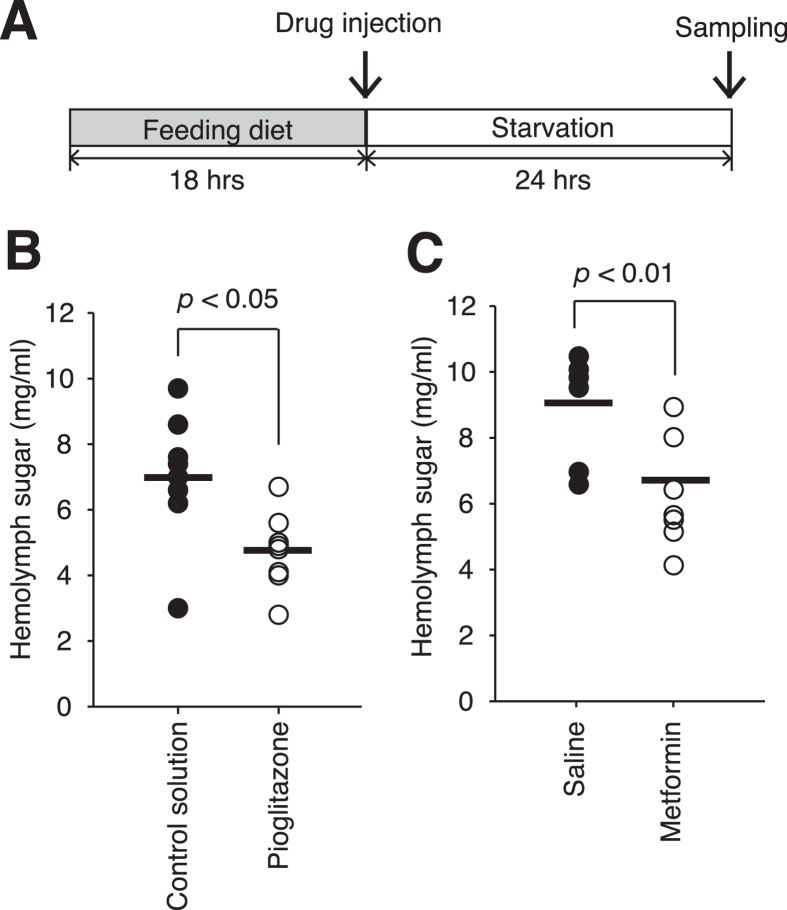
Decrease in fasting hemolymph sugar levels in the hyperlipidemic silkworm by administration of pioglitazone and metformin. (**A**) Experimental design. (**B**,**C**) Silkworms were fed a diet containing 10% (w/w) glucose (G.D.) for 18 h. (**B**) Pioglitazone (250 μg/larvae) or control solution (0.01 M HCl in PBS) was injected into the silkworm hemolymph. (**C**) Metformin (200 μg/larvae) or saline (0.9% NaCl) was injected into the silkworm hemolymph. Hemolymph sugar levels of the silkworms were determined after starvation for 24 h (n = 7-8/group). Bar represents mean. Significant differences between groups were evaluated using Student’s *t*-test.

**Figure 7:**
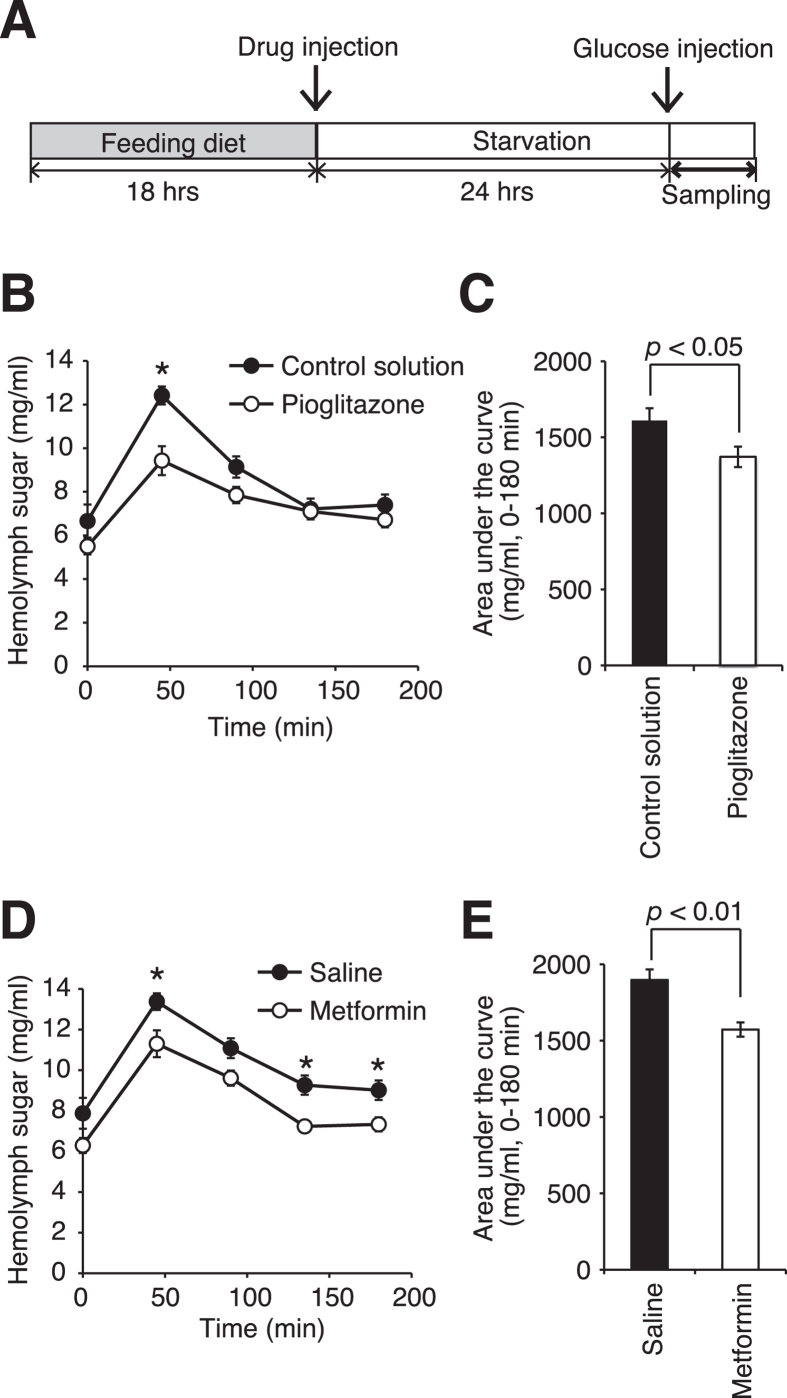
Improved glucose tolerance in the hyperlipidemic silkworm by administration of pioglitazone or metformin based on a glucose tolerance test. (**A**) Experimental design. (**B**,**C**) Silkworms were fed a diet containing 10% (w/w) glucose (G.D.) for 18 h, and then the diets were removed. (**B**) Pioglitazone (250 μg/larvae) or control solution (0.01 M HCl in PBS) was injected into the silkworm hemolymph. After incubation for 24 h, 100 μl of glucose (75 mg/ml) was injected into the silkworm hemolymph and hemolymph sugar levels were determined at 0, 45, 90, 135, and 180 min (n = 8/group). Data represent mean ± SEM. Asterisks indicate statistical significance based on Student’s *t*-test (*p* < 0.05). (**C**) Area under the curves of hemolymph sugar levels in Fig. 7B were calculated (n = 8/group). Data represent mean ± SEM. Significant differences between groups were evaluated using Student’s *t*-test. (**D**,**E**) Silkworms were fed a diet containing 10% (w/w) glucose (G.D.) for 18 h, and then the diets were removed. (**D**) Metformin (200 μg/larvae) or saline (0.9% NaCl) was injected into the silkworm hemolymph. After incubation for 24 h, 100 μl of glucose (75 mg/ml) was injected into the silkworm hemolymph and hemolymph sugar levels were determined at 0, 45, 90, 135, and 180 min (n = 7/group). Data represent mean ± SEM. Asterisks indicate statistical significance analyzed by Student’s *t*-test (*p* < 0.05). (**E**) Area under the curves of hemolymph sugar levels in Fig. 7D were calculated (n = 7/group). Data represent mean ± SEM. Significant differences between groups were evaluated using Student’s *t*-test.

Pioglitazone and metformin are clinically used for the treatment of type II diabetes^29^. We tested whether the hyperlipidemic silkworms would respond to these drugs. We fed silkworms a glucose-containing diet for 18 h, and then each drug was injected into the silkworm hemolymph (Fig. 6A). Administration of either pioglitazone or metformin reduced the fasting hemolymph sugar levels of the hyperlipidemic silkworms (Fig. 6B,C). We then tested whether the impaired glucose tolerance of the hyperlipidemic silkworms observed in the glucose tolerance test could be improved by injecting pioglitazone or metformin (Fig. 7A). The results demonstrated that hemolymph sugar levels of the hyperlipidemic silkworms pretreated with pioglitazone or metformin were significantly lower 45 min after the injection of glucose compared with silkworms injected with control solution (Fig. 7B,D). The area under the curve of the plots of the hyperlipidemic silkworms injected with pioglitazone or metformin was significantly smaller than that of the silkworms injected with control solution (Fig. 7C,E). These findings suggest that pioglitazone and metformin improved glucose tolerance in the hyperlipidemic silkworms.

## Discussion

In this study, we aimed to establish a type II diabetes model using silkworms for evaluating anti-diabetic drugs. We found that in silkworms, intake of a high-glucose diet for 18 h leads to the development of hyperlipidemia, insulin resistance, and impaired glucose tolerance. These characteristics of the hyperlipidemic silkworm resemble the symptoms of type II diabetes in humans. Moreover, administration of pioglitazone and metformin reduced the fasting hemolymph sugar levels of the hyperlipidemic silkworms. These findings support our notion that the hyperlipidemic silkworm model we established here could be regarded as a model of type II diabetes that is useful for evaluating the therapeutic activities of anti-diabetic drugs. We propose that this novel animal model will be useful for monitoring the therapeutic activities of candidate compounds for the treatment of type II diabetes.

We developed an *in vitro* insulin resistance test using fat bodies isolated from silkworms and a glucose tolerance test using individual silkworms. These assays may allow us to study the molecular mechanisms of insulin resistance and impaired glucose tolerance in the hyperlipidemic silkworms. Nonalcoholic fatty liver disease, in which triglycerides accumulate in the liver, is tightly associated with insulin resistance and is thus a marked feature of type II diabetes^28^. Continuous intake of a high calorie diet in mammals causes the accumulation of triglycerides in the liver and free fatty acids in the blood^7^. Further, the increase of free fatty acids in the blood activates JNK in the liver, leading to the development of insulin resistance^25^,^26^,^27^. We speculate that feeding silkworms a high-glucose diet for 18 h induces the accumulation of triglycerides in the fat bodies, which leads to an increase of free fatty acids in the hemolymph. The hyperlipidemic silkworm might thus also serve as a disease model of nonalcoholic fatty liver disease. Moreover, analyses of *jnk1* gene-knockout mice indicate that JNK in the cells of liver, adipose tissue, and muscle plays a critical role in adiposity and insulin resistance caused by feeding on a high-fat diet^30^. We assume that the accumulation of free fatty acids in the silkworm hemolymph activates JNK in the fat body cells, resulting in insulin resistance and finally in the development of impaired glucose tolerance. Recently, it was reported that fruit flies, *Drosophila melanogaster*, fed a glucose diet exhibit the accumulation of lipid in whole body homogenate along with insulin resistance^14^. Together with our present findings, we believe that the onset of diabetes induced by a high-calorie diet is a universal phenomenon that occurs not only in mammals, but also in insects such as fruit flies and silkworms. Previous studies suggest the association of hemolymph sugar level and development in insects. Trehalose and glucose levels in the hemolymph of the last larval instar of *Manduca sexta* changes depending on its developmental age^31^. *Drosophila melanogaster* larvae fed a high glucose diet result in the delay of pupalization^32^. We previously reported that silkworms fed a high glucose diet showed growth defect^24^. Thus, there is a possibility that the change of sugar levels observed in the hyperlipidemic silkworms is a result of delay in developmental age. We monitored the hemolymph sugar levels in silkworms fed a normal diet for 2 days and found that the sugar concentration does not change within this time-period (Supplementary Fig. 4). This result suggests that the sugar levels in hemolymph of silkworms do not change in our experimental schedule. Therefore, we consider that the changes observed in the hyperlipidemic silkworms could not be explained by the differences in developmental age.

In skeletal muscle cells, AMPK is shown to be activated by exercise and fuel deprivation, and its activation is related to glucose and fatty acid metabolisms^33^,^34^. Furthermore, phosphorylation of AMPK in cells of the skeletal muscle is an effective marker of type II diabetes^33^,^34^. In the hyperlipidemic silkworms, however, AMPK phosphorylation level was not significantly altered in the fat body compared to normal silkworms.

We found that the administration of pioglitazone and metformin led to a decrease in the hemolymph sugar levels of the hyperlipidemic silkworms after starvation for 24 h. To our knowledge, this is the first description of an invertebrate model animal suitable for evaluating the hypoglycemic actions of drugs for the treatment of type II diabetes. We performed an experiment to examine the phosphorylation level of JNK, AMPK, and Akt after injection of pioglitazone or metformin. The amounts of phosphorylated JNK, AMPK, and Akt in the fat bodies of hyperlipdemic silkworms 24 h post drug treatment were not significantly altered compared to control silkworms (Supplementary Fig. 5). The findings suggest that pioglitazone and metformin do not changes the JNK, Akt, and AMPK signaling pathway for 24 h after injection to hyperlipidemic silkworm. Pioglitazone, a thiazolidine derivative, improves insulin resistance by enhancing the functions of the transcription factor peroxisome proliferator-activated receptor-gamma (PPARγ)^35^. In mammals, PPARγ is highly expressed in adipose tissue as well as in the liver and skeletal muscle. The thiazolidine derivatives act on various organs and reduce blood glucose levels. In adipose tissue, thiazolidine derivatives induce an increase in the number of small adipocytes that are sensitive to insulin; upregulate the expression of adiponectin, which is involved in the regulation of blood glucose level; and enhance fatty acid uptake. In skeletal muscle, thiazolidine derivatives upregulate the expression of PPARγ, stimulate sugar uptake, and enhance glycogen synthesis. In the liver, thiazolidine derivatives inhibit gluconeogenesis. Several thiazolidinediones and their derivatives were identified as inhibitors of mitochondrial pyruvate carrier (MPC), which is essential for mitochondrial pyruvate transport^36^,^37^,^38^. MPC is highly conserved among yeast, drosophila, and human and is required for pyruvate metabolism^39^,^40^. Pyruvate is the end-product of glycolysis involved in the metabolism of lipid, amino acids, and glucose^41^. A recent study demonstrated that mild MPC inhibition by pioglitazone increased plasma membrane glucose uptake in C2C12 myoblasts^37^. We therefore speculate that pioglitazone may also act as an inhibitor of MPC in silkworms and enhance plasma membrane sugar uptake. On the other hand, metformin, a biguanide agent, contributes to the improvement of diabetic conditions by activating AMPK in the liver cells and skeletal muscle cells of mammals^42^. Furthermore, a recent study showed that metformin directly inhibits the enzyme activity of mitochondrial glycerophosphate dehydrogenase leading to the suppression of gluconeogenesis^43^. Therefore, we assume that metformin acts as an inhibitor of mitochondrial glycerophosphate dehydrogenase in cells of various organs of the silkworm. This action of metformin may contribute to the decrease in hemolymph sugar level of the hyperglycemic silkworms.

In conclusion, this silkworm model mimicking type II diabetes is useful for the evaluation of anti-diabetic drugs that are effective for type II diabetes, and may potentially contribute to the discovery of new anti-diabetic drugs.

## Methods

### Silkworm rearing conditions, glucose diet preparation, and injection methods

Silkworms were raised from fertilized eggs (Hu·Yo x Tukuba·Ne; Ehime Sanshu) to fifth-instar larvae. The fifth-instar larvae were fed a diet containing 10% glucose (high-glucose diet) for 18 h. The high-glucose diet was prepared by mixing Silkmate 2S (Nosan Corporation) and D-glucose. Injection experiments were performed as previously described^18^. The test sample (50 μl) was injected into the silkworm hemolymph through the dorsal surface using a 27-gauge needle.

### Determination of hemolymph sugar levels

Hemolymph sugar levels were determined by the previously described method^24^. Hemolymph (5 μl) was collected from the silkworms through a cut on the first proleg, and immediately mixed with 9 volumes of 0.6 N perchloric acid. The supernatant, after centrifugation at 15,000 rpm (20,400 g) for 3 min, was appropriately diluted with distilled water for sugar determination. Total sugar in the hemolymph was determined using the phenol-sulfuric acid method^44^. Serially diluted D-glucose solution was used as a standard.

### Determination of fat body glycogen amounts

The fat body was isolated from the dorsolateral region of each larvae, and rinsed in insect saline (10 mM Tris/HCl, 130 mM NaCl, 5 mM KCl, and 1 mM CaCl_2_). The fat body (wet weight 5~20 mg) was lysed in 50 μl of 30% KOH with boiling for 10 min. Distilled water (150 μl) and ethanol (300 μl; final 60%) were added and the mixture was boiled for 10 min. The samples were incubated at 4 °C overnight and centrifuged at 15,000 rpm for 3 min. The precipitate was dissolved in distilled water to give a concentration of 100 mg fat body/ml by boiling for 10 min. The resulting fat body extract was used for sugar quantification by the anthrone-sulfuric acid method^45^. Serially diluted D-glucose solution was used as a standard. The amount of sugar in 1 mg of fat body was calculated.

### Determination of triglyceride and free fatty acid amounts

The fat body (wet weight 1~10 mg) was rinsed in insect saline (10 mM Tris/HCl, 130 mM NaCl, 5 mM KCl, and 1 mM CaCl_2_). The amounts of triglycerides and free fatty acids were determined using quantification kits based on enzymatic reaction. Triglyceride levels were measured using Triglyceride E-test Wako (Wako). Free fatty acid levels were measured using NEFA C-test Wako (Wako).

### Chemicals

Recombinant human insulin was purchased from Wako and dissolved in 0.9% NaCl containing 0.1% acetic acid. Pioglitazone was purchased from LKT Laboratories. Five milligrams of pioglitazone was dissolved in 100 μl of 0.1 M HCl, and boiled for 4 min. The solution was added to 900 μl of phosphate buffered saline (PBS) and mixed by vortex. Metformin (1,1-dimethylbiguanide hydrochloride) was purchased from Wako and dissolved in 0.9% NaCl.

### Oil red O staining of the silkworm fat bodies

The isolated fat bodies were rinsed twice in insect saline (10 mM Tris/HCl, 130 mM NaCl, 5 mM KCl, and 1 M CaCl_2_) and then fixed in 3.7% formaldehyde at room temperature for 30 min. The fat bodies were treated with 60% isopropanol for 1 min and then transferred to Oil red O stain solution (1.8 mg/ml) and incubated at room temperature for 20 min. After incubation, the fat body samples were rinsed 5 times in 60% isopropanol. The dried fat body samples were immersed in 200 μl xylene and sonicated. The fat body samples were centrifuged at 10,000 rpm for 3 min and absorbance of the supernatant was measured at 490 nm. The absorbance per gram of fat body was calculated.

### Immunoblot analysis

Western blot analysis for detection of proteins in the silkworm fat bodies was performed as previously described^24^. Briefly, fat bodies isolated from the dorsolateral region of the larvae were rinsed in insect saline (10 mM Tris/HCl, 130 mM NaCl, 5 mM KCl, and 1 mM CaCl_2_) and then transferred to NP-40 lysis buffer (10 mM Tris/HCl [pH 7.5], 150 mM NaCl, 0.5 mM EDTA, 1 mM dithiothreitol, 1% NP-40, 10 mM NaF, and 1 mM Na_3_VO_4_), and lysed by sonication using Sonifier 450 (Branson). The mixture was precipitated with 5% trichloroacetic acid, electrophoresed in a 12.5% polyacrylamide gel, and electroblotted onto a polyvinylidene difluoride membrane (Millipore), probed with antibody, and detected using Western Lightning (Perkin-Elmer Life Sciences). The following antibodies were used for immunoblot analysis: rabbit polyclonal antibodies to, phosphorylated Akt (Cell Signaling), phosphorylated JNK (Promega), phosphorylated AMPK (Cell Signaling) and β-actin (Cell Signaling). Quantification of the amount of phosphorylated JNK, phosphorylated Akt, or phosphorylated AMPK was performed by densitometric scanning with Image Gauge software. The relative amount of phosphorylated JNK or phosphorylated Akt or phosphorylated AMPK to β-actin was determined.

### Glucose tolerance test

Silkworms were fed a high-glucose diet for 18 h, and test sample (50 μl) was injected into the hemolymph of the silkworms (body weight 1.40~1.55 g). After injection, the silkworms were reared without food for 24 h. One hundred microliters of glucose solution (75 mg/ml) was injected into the hemolymph, and the hemolymph was subsequently collected at the indicated time points for sugar quantification. The area under the curve was calculated from the plots of the hemolymph sugar levels of each silkworm.

### Statistical Analysis

Data are shown as means ± standard error of the mean (SEM). Significant differences between groups were evaluated using a two-tailed Student’s *t* test. A p-value of less than 0.05 was considered statistically significant.

## Additional Information

**How to cite this article**: Matsumoto, Y. *et al.* Diabetic silkworms for evaluation of therapeutically effective drugs against type II diabetes. *Sci. Rep.* **5**, 10722; doi: 10.1038/srep10722 (2015).

## Supplementary Material

Supplementary Information
