# Supplementary material for: Diabetic silkworms for evaluation of therapeutically effective drugs against type II diabetes
Source: Sci Rep. 2015 May 29;5:10722. doi: 10.1038/srep10722 (PMC4448660; doi:10.1038/srep10722)
Supplement: Supplementary Information [file srep10722-s1.pdf]

Supplementary information

Diabetic silkworms for evaluation of therapeutically effective drugs against type II diabetes.

Yasuhiko Matsumoto, Masaki Ishii, Yohei Hayashi, Shinya Miyazaki, Takuya Sugita, Eriko Sumiya, and Kazuhisa Sekimizu

[1]*Laboratory of Microbiology, Graduate School of Pharmaceutical Sciences, The University of Tokyo, 7-3-1 Hongo, Bunkyo-ku, Tokyo 111-0033, Japan.*

Correspondence to: Dr. Kazuhisa Sekimizu, Laboratory of Microbiology, Graduate School of Pharmaceutical Sciences, The University of Tokyo, 7-3-1 Hongo, Bunkyo-ku, Tokyo 113-0033, Japan; sekimizu@mol.f.y-tokyo.ac.jp

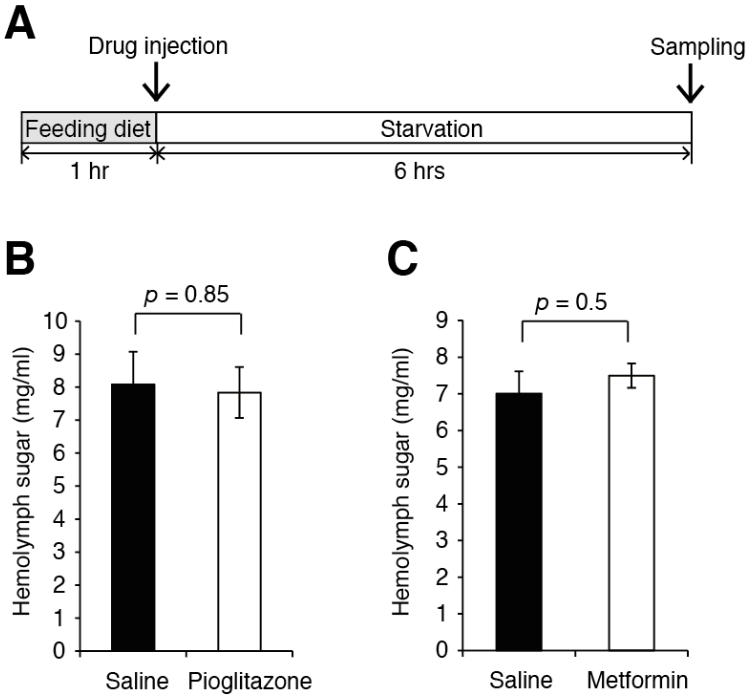

Supplementary figure 1 Effects of pioglitazone and metformin on hemolymph sugar levels in silkworms made hyperglycemic by intake of a high-glucose diet for 1 h. (A) Experimental design. (B, C) Silkworms were fed a diet containing 10% (w/w) glucose (G.D.) for 1 h. (B) Pioglitazone (100 µg/larvae) or saline (0.9% NaCl) was injected into the silkworm hemolymph. (C) Metformin (100 µg/larvae) or saline (0.9% NaCl) was injected into the silkworm hemolymph. Hemolymph sugar levels of the silkworms were determined after starvation for 6 h (n = 4-5/group). Data represent mean ± SEM. Significant differences between groups were evaluated using Student's *t*-test.

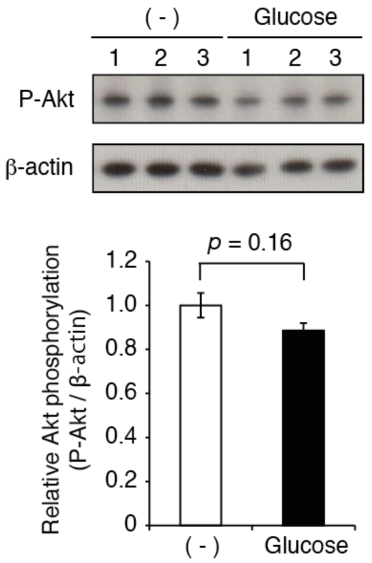

Supplementary figure 2 Effect of high glucose on Akt phosphorylation in the fat bodies of silkworms *in vitro*. Silkworms were fed a normal diet (N.D.) for 18 h, and then the fat bodies were isolated. Fat bodies were cultured in Grace's insect medium with or without glucose (350 mg/dL) at 27°C for 3 h (n = 3/group). Phosphorylated Akt and β-actin were determined by Western blot analysis (Top). Samples were loaded in the same gel. Cropped blots were used. Relative amount of phosphorylated Akt was determined (Bottom). Data represent mean ± SEM. Significant differences between groups were evaluated using Student's *t*-test.

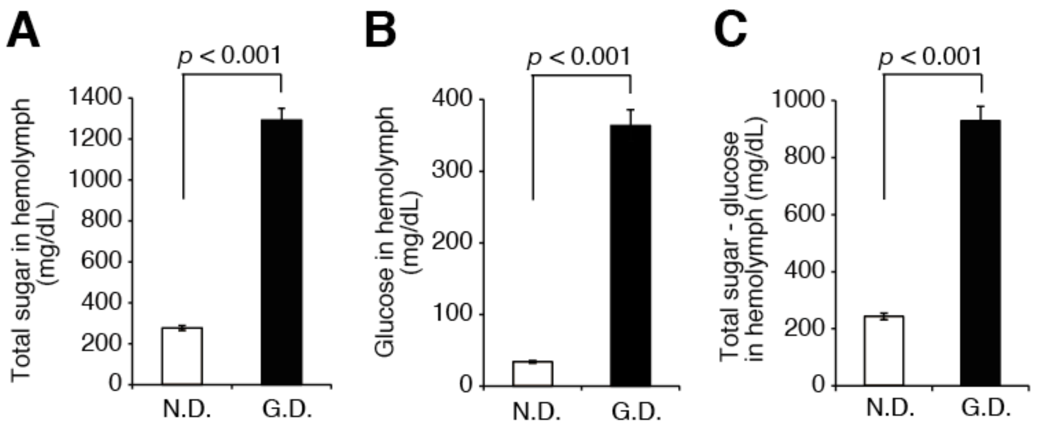

Supplementary figure 3 Increases in glucose levels in the hyperlipidemic silkworm. (A-C) Silkworms were fed a normal diet (N.D.) or a diet containing 10% (w/w) glucose (G.D.) for 18 h. (A) Total sugar levels in hemolymph of the silkworms were determined using the phenol-sulfuric acid method (n = 7/group). (B) Glucose levels in hemolymph of the silkworms were determined using a glucometer (Accu-Chek Aviva, Roche) (n = 7/group). (C) Non-glucose sugar levels in hemolymph of the silkworms were determined by subtracting the amount of glucose from total sugar (n = 7/group). Data represent mean ± SEM. Statistical significance between groups were evaluated using Student's *t*-test.

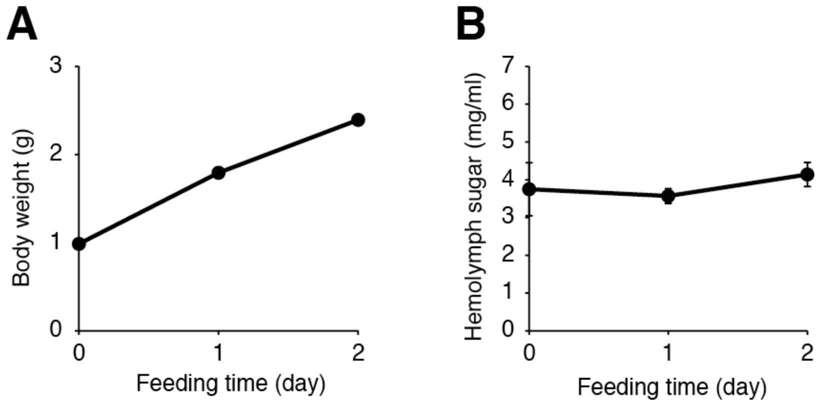

Supplementary figure 4 Relation of developmental age and hemolymph sugar level in silkworms. (A and B) Silkworms were fed a normal diet (N.D.) for 2 days. (A) Body weight of the silkworms were determined (n = 3/group). (B) Total sugar levels in hemolymph of the silkworms were determined using the phenol-sulfuric acid method (n = 3/group). Data represent mean ± SEM.

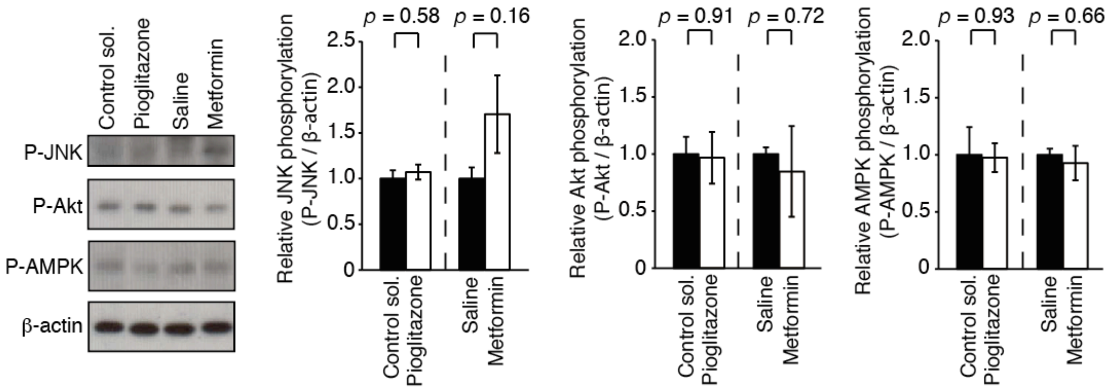

Supplementary figure 5 Effects of pioglitazone and metformin against JNK, Akt, AMPK signaling pathway. Silkworms were fed a diet containing 10% (w/w) glucose (G.D.) for 18 h. Pioglitazone (250 µg/larvae) or control solution (0.01 M HCl in PBS) was injected into the silkworm hemolymph. Metformin (200 µg/larvae) or saline (0.9% NaCl) was injected into the silkworm hemolymph. Fat bodies of the silkworms were isolated at 24 h after injection (n = 4/group). Phosphorylated JNK, phosphorylated Akt, phosphorylated AMPK and β-actin were determined by Western blot analysis. Samples were loaded in the same gel. Cropped blots were used. Relative amounts of phosphorylated JNK, phosphorylated Akt, and phosphorylated AMPK were determined. Data represent mean ± SEM. Significant differences between groups were evaluated using Student's *t*-test.

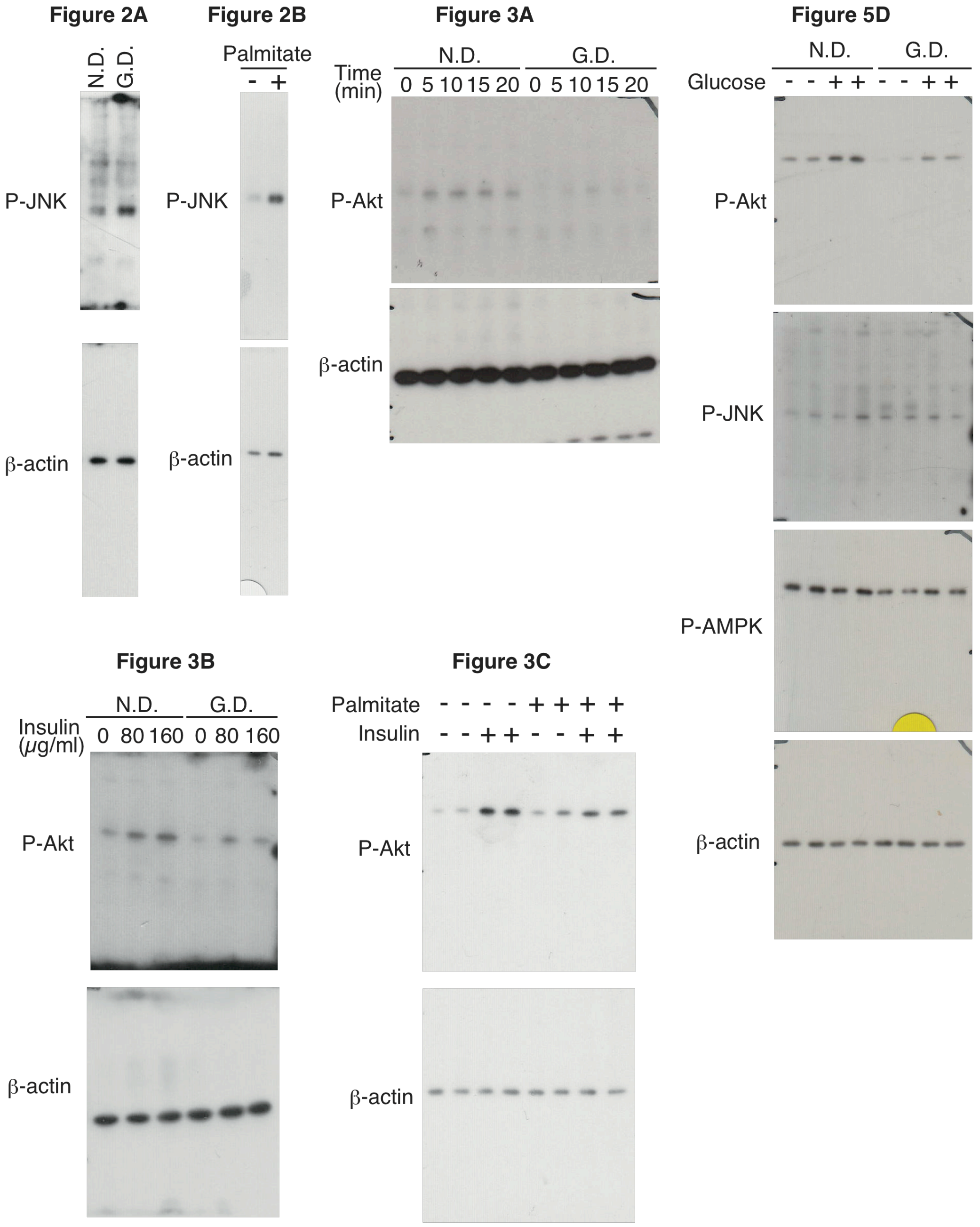

Supplementary figure 6 Full-length blots of Figure 2, 3, and 5D.
